# Synthetic Flavonoids as Novel Modulators of Platelet Function and Thrombosis

**DOI:** 10.3390/ijms20123106

**Published:** 2019-06-25

**Authors:** Thomas M. Vallance, Divyashree Ravishankar, Dina A. I. Albadawi, Helen M. I. Osborn, Sakthivel Vaiyapuri

**Affiliations:** School of Pharmacy, University of Reading, Whiteknights, Reading RG6 6UB, UK; t.m.vallance@pgr.reading.ac.uk (T.M.V.); divyasri.april86@gmail.com (D.R.); d.a.i.albadawi@pgr.reading.ac.uk (D.A.I.A.); h.m.i.osborn@reading.ac.uk (H.M.I.O.)

**Keywords:** platelets, flavonoids, thrombosis, haemostasis, synthetic flavonoids, bioavailability

## Abstract

Cardiovascular diseases represent a major cause of mortality and morbidity in the world, and specifically, thrombotic conditions such as heart attacks and strokes are caused by unwarranted activation of platelets and subsequent formation of blood clots (thrombi) within the blood vessels during pathological circumstances. Therefore, platelets act as a primary therapeutic target to treat and prevent thrombotic conditions. Current treatments are limited due to intolerance, and they are associated with severe side effects such as bleeding complications. Hence, the development of novel therapeutic strategies for thrombotic diseases is an urgent priority. Flavonoids are naturally occurring plant-derived molecules that exert numerous beneficial effects in humans through modulating the functions of distinct cell types. However, naturally occurring flavonoids suffer from several issues such as poor solubility, lipophilicity, and bioavailability, which hinder their efficacy and potency. Despite these, flavonoids act as versatile templates for the design and synthesis of novel molecules for various therapeutic targets. Indeed, several synthetic flavonoids have recently been developed to improve their stability, bioavailability, and efficacy, including for the modulation of platelet function. Here, we provide insight into the actions of certain natural flavonoids along with the advantages of synthetic flavonoids in the modulation of platelet function, haemostasis, and thrombosis.

## 1. Introduction

Cardiovascular diseases (CVDs) are the biggest cause of death in humans [1,2]. Among various CVDs, thrombotic diseases such as heart attacks and strokes affect a large proportion of people. In 2017, in the UK alone, approximately 2.3 million people lived with coronary heart disease, 1.4 million experienced a heart attack, and 1.3 million suffered from a stroke or transient ischaemic attack [2]. Currently used antiplatelet/antithrombotic therapies help to save lives although they have numerous adverse side effects such as uncontrolled bleeding, and many patients do not respond to these treatments [3,4]. Therefore, novel therapeutic strategies to treat and prevent thrombotic diseases are urgently needed to reduce the burden and cost placed on healthcare services worldwide. Natural resources such as plants and microbes have provided inspiration for the development and production of new drugs over a sustained period, with examples including aspirin, fentanyl, and penicillin. Following on from this traditional development, flavonoids have been suggested as key underexploited natural resources for drug development [5,6].

Platelets are small circulating blood cells that are responsible for maintaining haemostasis via blood clotting upon vascular injury. Although they are anucleated cells, they are derived from megakaryocytes and possess all the organelles and proteome required for normal cellular function [7,8]. Alongside their role in the maintenance of haemostasis, due to their high number in circulation, platelets act as immune sentinels and protect against invading microorganisms via response to and production of pro- and anti-inflammatory molecules [7,9]. Notably, platelets are the major players in the development of CVD, specifically, thrombotic diseases such as ischaemic stroke and myocardial infarction. Thrombotic diseases occur when the platelets are activated unnecessarily under pathological conditions, and as a result, the blood clots formed within the blood vessels reduce/occlude the blood flow to major organs such as the brain or heart and induce ischaemia and necrosis of the tissue [3].

Due to the presence of a repertoire of receptors and proteins on the surface of platelets, they respond to various internal and external stimuli. The activation of platelets in response to blood vessel damage has been described in detail elsewhere [7,8,9,10,11]. Briefly, platelets respond to the exposure of collagen when the blood vessel wall is damaged via glycoprotein (GP) Ib/V/IX (ligates von Willebrand Factor (vWF)) and subsequently GPVI (ligates collagen) and form a monolayer at the injury site. This promotes the secretion of platelet granules and activation of further platelets in circulation via secondary activators such as adenosine diphosphate (ADP) and thromboxane A_2_ (TxA_2_). Platelets associate via fibrinogen, which binds to integrin α_IIb_β_3_, and this binding can induce outside-in signalling via this integrin and results in stabilisation of the clot [7,8,9,10,11,12].

**Figure 1:**
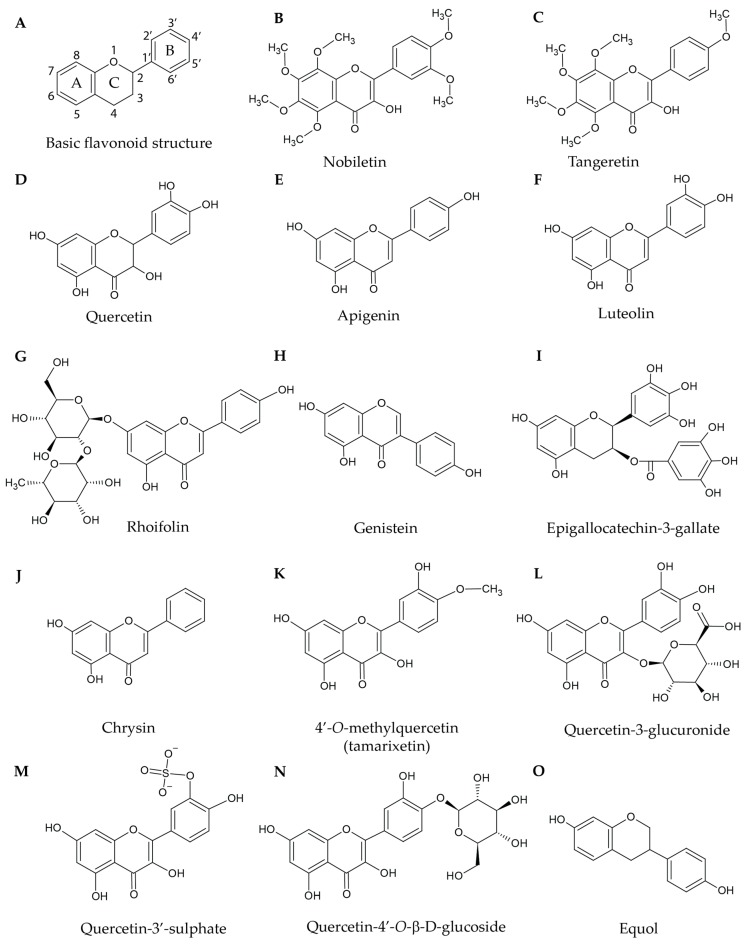
Structures of selected natural flavonoids and metabolites: (**A**) The basic structure of flavonoids. A, B, and C denote the A-ring, B-ring, and C-ring respectively. Nobiletin (**B**) and tangeretin (**C**) are both heavily methoxylated. Quercetin (**D**), apigenin (**E**), and luteolin (**F**) possess similar structures but differ in the extent of their hydroxylation. Rhoifolin (**G**) is a flavonoid with a glycoside bond at C7 on the A-ring. Genistein (**H**) has a simple structure similar to apigenin; however, the B-ring is connected at C3 rather than C2. Epigallocatechin-3-gallate (**I**) possesses an ester bond connecting the phenyl ring to the flavonoid core. Chrysin (**J**) is a very simple flavonoid with no hydroxyl groups attached to the phenyl B-ring. 4′-*O*-methylquercetin (**K**), quercetin-3-glucuronide (**L**), and quercetin-3′-sulphate (**M**) are metabolites of quercetin and are either methylated, glucuronidated, or sulphated, respectively. Quercetin-4′-*O*-β-d-glucoside (**N**) has a glucosidic bond at the 4′ position. Equol (**O**) is a metabolite that is produced by gut bacteria.

Flavonoids (Figure 1A) are naturally occurring yet structurally diverse compounds that share a common core and are found primarily in plants in which they have multiple roles. They are regularly consumed as part of a routine diet. In plants, they act as antioxidants to remove free radicals and to aid in growth and development; some are involved in immune defence due to their ability to act as antimicrobial compounds [5,6]. Furthermore, due to their ultraviolet radiation-protective properties, flavonoids have been hypothesised to have been critical for the transition of plants to the land [6]. Indeed, flavonoids are highly noticeable in plants because they also function as attractants for pollinators and seed dispersors by providing colour to flowers and fruits. However, as flavonoids are present in all plant structures, they are also available for consumption by humans and other animals [13]. As flavonoids have been shown to possess many beneficial effects for humans including within disease states, they have been identified as potential candidates for synthetic modifications for the development of novel therapeutic agents. Despite their beneficial effects, natural flavonoids lack sufficient bioavailablity, solubility, stability, lipophilicity, and efficacy. In addition, they have multiple targets in cellular systems. However, the ability to modify the structures of naturally occurring flavonoids has led to the development of synthetic compounds with improved biological characteristics for the treatment of multiple ailments via diverse mechanisms. These modifications also promote specificity to the desired molecular target, improve the bioavailability, and increase resistance to first-pass metabolism. Flavonoids have already yielded a source of pharmacological compounds such as LY294002, a phosphoinositide-3-kinase (PI3K) inhibitor based on the structure of quercetin [14].

Here, we briefly review the current knowledge on the impact of naturally occurring flavonoids on the modulation of platelet function and illustrate how synthetic flavonoids with improved efficacy are being developed, providing a potential platform for the development of new therapeutics for CVD (especially thrombotic diseases).

## 2. Naturally Occurring Flavonoids

Numerous naturally occurring flavonoids have been tested extensively on the modulation of platelet activation, thrombosis, and haemostasis. The previous studies have provided detailed information on the ability of naturally occurring flavonoids to affect the diverse functions of platelets. Moreover, some of these studies have identified specific molecular targets for the flavonoids in platelets. Interestingly, some studies demonstrate that flavonoids have many targets both on the platelet surface and intracellularly, as flavonoids have been shown to be internalised in platelets [15,16]. Here, we briefly discuss the impact of specific naturally occurring flavonoids on the modulation of platelet function. 

### 2.1. Flavanoids Affect Platelet Function

Most studies demonstrate the effects of flavonoids on key platelet outputs, specifically platelet aggregation in response to multiple agonists such as collagen, ADP, U46619, and thrombin. Notably, collagen can activate two distinct receptors (GPVI and integrin α_2_β_1_), and therefore, synthetic cross-linked collagen-related peptide (CRP-XL) has been developed to selectively activate platelets via GPVI [8,17,18]. From our research group, nobiletin (Figure 1B) and tangeretin (Figure 1C), two structurally similar naturally occurring flavonoids found in citrus fruits, particularly lemons, have been examined for their antiplatelet effects [19,20]. These two compounds were tested for their effects on collagen- and CRP-XL-induced platelet aggregation, and they both were effective at inhibiting aggregation in a concentration-dependent manner [19,20]. Similarly, many more structurally diverse flavonoids such as quercetin (the most abundant flavonoid in human diets; Figure 1D; IC_50_ = 58.6 ± 6.5 μM [21]), apigenin (Figure 1E; IC_50_ = 9.3 ± 0.9 μM), luteolin (Figure 1F; IC_50_ = 40 ± 13.3 μM), rhoifolin (Figure 1G; IC_50_ = 425.1 ± 224.6 μM), genistein (Figure 1H; IC_50_ = 11.9 ± 1.5 μM), epigallocatechin-3-gallate (EGCG; Figure 1I), and chrysin (Figure 1J) have been shown to inhibit collagen-induced platelet aggregation at distinct IC_50_ concentrations [15,22,23,24,25,26]. Metabolites of quercetin (4′-*O*-methylquercetin (tamarixetin; Figure 1K), quercetin-3-glucuronide (Figure 1L), and quercetin-3′-sulphate (Figure 1M)) displayed a reduced ability to inhibit collagen-induced platelet aggregation compared with quercetin itself [15]. This data suggests that the addition of large or charged functional groups decreases the efficacy of flavonoids and may be due to glucuronidation and sulphation (which are common modifications occurring in the intestine) affording metabolites that are too hydrophilic to cross cell membranes [15,27].

**Figure 2:**
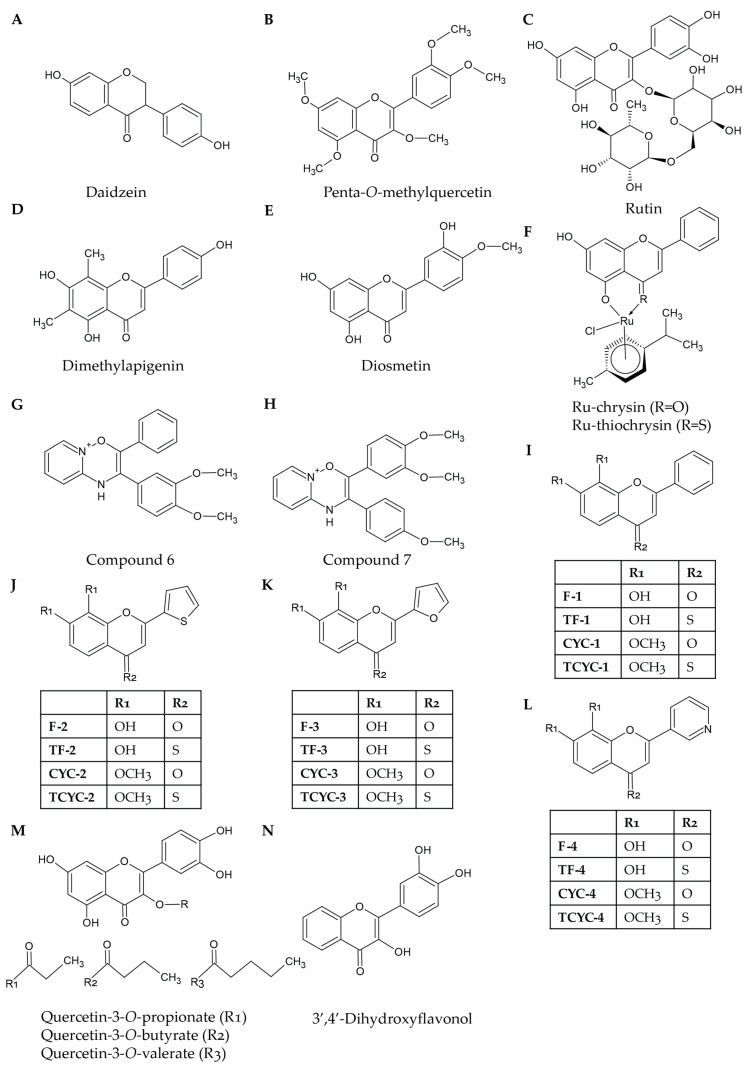
Structures of selected natural and synthetic flavonoids: Daidzein (**A**) is the flavonoid from which equol is derived. Penta-*O*-methylquercetin (**B**) has a similar structure to quercetin; however, all of its hydroxyl groups have been methylated. Like rhoifolin, rutin (**C**) also has a glycosidic bond; however, in this compound, it is connected at C3 as opposed to C7. Dimethylapigenin (**D**) is structurally similar to apigenin; however, it possesses two methyl groups at the C6 and C8 positions. Diosmetin (**E**) and luteolin have similar structures; however, the 4′-hydroxyl group in luteolin has been replaced with a methoxy group in diosmetin. Ru-chrysin and Ru-thiochrysin (**F**) share the same structure as chrysin; however, they have been conjugated to ruthenium. The two compounds differ due to the substitution of a carbonyl group with a thiol group attached to C4. Compound **6** (**G**) and compound **7** (**H**) are pyrido(1,2-*a*)pyrimidin-4-one analogues that possess methoxylated phenyl groups attached to C2 and C3. Ravishankar et al. [35] developed hydroxylated or methoxylated synthetic flavonoids with modified B-rings. The B-rings could be either phenyl groups (**I**), thiofuran groups (**J**), furan groups (**K**), or pyridyl groups (**L**). Synthetic modifications of flavonoids can lead to the addition and extension of acyl chains at C3 to molecules such as quercetin (**M**). 3′,4′-Dihyrdoxyflavonol (**N**) is a synthetic flavonoid based on the structure of a flavonol but with two hydroxyl groups at the C3′ and C4′ positions.

Following the initial activation of platelets, they release multiple secondary activators and signalling molecules in order to activate and recruit further circulating platelets, to trigger several signalling cascades, and to increase thrombus size [8]. This later stage of platelet activation is mainly mediated by molecules such as ADP and TxA_2_ [8,10]. Flavonoids have been shown to be effective at inhibiting isolated platelet aggregation mediated by these molecules. Specifically, apigenin (IC_50_ = 34.6 ± 13.4 μM), luteolin (IC_50_ = 56.9 ± 20.8 μM), genistein (IC_50_ = 24.5 ± 7.0 μM), and quercetin (IC_50_ = 60.6 ± 16.8 μM) were capable of inhibiting platelet aggregation induced by arachidonic acid (AA; a substrate of cyclooxygenase-1 (COX-1) [28]) [22]. These same flavonoids were also capable of inhibiting platelet aggregation induced by U46619 (a synthetic TxA_2_ mimetic [29]): apigenin (IC_50_ = 23.8 ± 5.9 μM), luteolin (IC_50_ = 47.0 ± 13.3 μM), rhoifolin (IC_50_ = 398.0 ± 165.4 μM), genistein (IC_50_ = 24.7 ± 10.9 μM), and quercetin (IC_50_ = 74.5 ± 14.4 μM) [30]. However these IC_50_ concentrations are greater than the concentrations that can be acheived in human blood (e.g., 10.66 μM for quercetin whereas apigenin was only detected at a concentration of 0.1 μM) [31]. Equol (Figure 1O) is a metabolite of daidzein (Figure 2A) produced by gut bacteria and it inhibits aggregation of isolated platelets induced by ADP and U46619 [32]. This inhibition was achieved at physiologically relevant concentrations; however, it has been shown that daidzein is not converted to equol in every member of the general population, possibly due to differences in gut microbiota [32,33,34].

Several complex yet naturally occurring flavonoids isolated from *Justicia procumbens* and *Cephalotxus wilsoniana* were assayed for their effects on platelet-rich plasma (PRP) stimulated with adrenaline and were demonstrated to be able to acutely supress adrenaline-induced platelet activation [36]. Furthermore, chrysin (by approximately 75%) and purple grape juice (by approximately 50%) have been shown to decrease platelet aggregation induced by ADP or U46619 in PRP [25,37].

Moreover, thrombin is a protease generated on the surface of activated platelets that can stimulate platelets via the cleavage of extracellular portions of protease-activated receptor 1 (PAR1) and PAR4 [10]. Some flavonoids were demonstrated to inhibit thrombin-induced platelet aggregation; however, the concentrations required are greater than those necessary for collagen-induced platelet aggregation (e.g., 150 μM nobiletin for thrombin versus 25 μM nobiletin for collagen) [19,20,25,38,39,40,41]. This is mainly due to the fact that thrombin is a more potent agonist than collagen.

Most studies researching flavonoids have investigated their acute effects in *ex vivo* conditions; however, a few flavonoids have been tested for their efficacy under *in vivo* settings. In humans, quercetin 4′-*O*-β-d-glucoside (Q-4-G; Figure 1N) has been shown to inhibit low-dose collagen-induced isolated platelet aggregation when volunteers were given a Q-4-G supplemented drink orally either 30 min or 120 min beforehand, and oral consumption of quercetin-rich onion soup in humans has also resulted in diminished agonist-induced platelet activation [42]. Furthermore, tail bleeding experiments can be used to determine the effects of flavonoids on haemostasis. Nobiletin, chrysin, and tangeretin were seen to prolong the bleeding time of mice in a tail bleeding assay when infused intravenously (IV), e.g., 200 s to 252 s with 10 μM tangeretin [19,20]. Ferric chloride-induced carotid arterial injury is a common model used to investigate the effects of antiplatelet drugs on occlusive thrombosis [43]. Penta-*O*-methylquercetin (PMQ; Figure 2B; a methylated version of quercetin) was found to prevent occlusive thrombosis in mice when given IV 30 min prior to the induction of a ferric chloride injury [40]. Mosawy et al. [39] investigated the effect of quercetin 24 h after a 7-day course of intraperitoneal (IP) treatment [39]. They demonstrate that quercetin was cardioprotective as blood flow was unchanged 15 min after the ferric chloride injury; however, this was most effective when a single IV bolus was used rather than a 7-day course of IP injections [39]. Further work is required to better understand the pharmacokinetic profile of the various compounds in order to improve their retention in the bloodstream.

### 2.2. Flavonoids Affect Specific Targets in Platelets

Calcium mobilisation is required in platelets for activation as its entry into the cytoplasm from intracellular stores enables its binding to calcium-dependent protein kinase (PKC), which has multiple effects in platelet activation [10,44]. Flavonoids such as EGCG and nobiletin have been shown to inhibit calcium mobilisation induced by collagen or CRP-XL in a dose-dependent manner, e.g., up to 83.5% following EGCG treatment [19,23]. Furthermore, apigenin, genistein, quercetin, and catechin have been shown to inhibit calcium mobilisation induced by a low dose of thrombin (0.2 U/mL), ADP, collagen, and U46619 [30,38,45,46]. These studies indicate that calcium mobilisation may be affected by different flavonoids at distinct stages, thereby inhibiting processes that require calcium such as phosphatidylserine (PS) exposure [45]. As platelets are activated, their surfaces become enriched in PS to enable the generation of thrombin [11]. Quercetin and catechin were demonstrated to inhibit the exposure of PS induced by a range of platelet agonists in a dose-dependent manner (25–100 μM for quercetin and 50–100 μM for catechin), which in turn led to a significant decrease in thrombin generation [45].

Fibrinogen binding is a useful measure of platelet activation as it can be used as a marker for inside-out signalling to integrin α_IIb_β_3_ [18]. During activation of platelets, there is inside-out activation of integrin α_IIb_β_3_ which enables it to bind to fibrinogen and subsequently trigger aggregation, and outside-in signalling at a later stage to promote clot retraction [12]. Clot formation is mediated by fibrinogen as it is acting as a molecular scaffold. The outside-in signalling is important for normal platelet function as it enables platelet spreading during monolayer formation as well as clot retraction to promote wound healing [12]. The outside-in signalling through integrin α_IIb_β_3_ is affected by flavonoids such as nobiletin and tangeritin as the clot weight increased, and thus, clot retraction was diminished [19,20]. Chrysin inhibits clot retraction as well as the phosphorylation of signalling proteins such as spleen tyrosine kinase (Syk) and phospholipase Cγ2 (PLCγ2) which are involved in platelet activation [12,25]. Furthermore, platelet spreading on collagen and fibrinogen surfaces was reduced by the presence of quercetin whilst chrysin inhibits spreading on fibrinogen [21,25].

**Figure 3:**
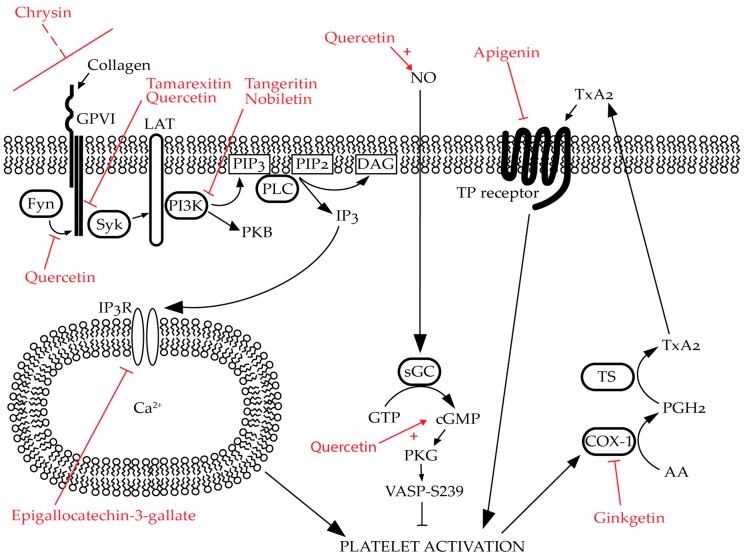
Predicted/established targets of selective flavonoids in platelets: Flavonoids modulate platelet activation through multiple mechanisms. For example, quercetin inhibits Fyn and Syk phosphorylation whilst also promoting nitric oxide (NO) generation in endothelial cells. Tamarexitin inhibits Syk phosphorylation. Chrysin inhibits glycoprotein (GP)VI-mediated signalling; however, the mechanism of action for this is unknown. Tangeretin and nobiletin inhibit phosphoinositide 3-kinase (PI3K) whilst nobiletin also promotes the generation of cyclic guanosine monophosphate (cGMP). Epigallocatechin-3-gallate inhibits calcium (Ca^2+^) mobilisation by promoting the inhibitory phosphorylation of inositol trisphosphate receptor (IP_3_R). Apigenin inhibits thromboxane A_2_ (TxA_2_) signalling by antagonising the thromboxane A_2_ (TxA_2_) receptor (TP receptor) whilst ginkgetin inhibits the same pathway by reversibly inhibiting COX-1. Please note that these flavonoids may have additional targets beyond those shown in this figure, and these are only a selected examples. Flavonoids are highlighted in red. Naturally occuring signalling pathways are represented by black arrows. “T” arrows denote inhibition whether it occurs naturally (black) or due to the effects of flavonoids (red). Red, dashed line indicates uncertainty in the specific target of the flavonoid. Red arrows coupled with red pluses denote potentiation of activity evoked by the flavonoid.

The TxA_2_ receptor (TP receptor; Figure 3) is a target for flavonoids as apigenin, genistein, daidzein, luteolin, and quercetin displace radiolabelled TP receptor antagonists, which suggests that their inhibition is partially due to antagonism of the TP receptor. This has been proposed to be due to similarities in the shapes between apigenin, luteolin, genistein, and TxA_2_ molecules [22,30,32]. This study has also found that methylation may decrease the affinity of the flavonoids for the receptor [22]. Furthermore, equol has been shown in platelets to compete for binding at TP receptors with a higher affinity than its parent molecule, daidzein (0.581 ± 0.062 nM vs. 63.44 ± 5.73 nM) [32].

In contrast, the binding of thrombin to platelets is unaffected by the presence of flavonoids, which suggests that their effects are mediated intracellularly and not by direct receptor antagonism [38]. Flavanoids are well-known to enter into intracellular regions. One protein that has been strongly linked to the mechanisms of action of flavonoids is cyclooxygenase-1 (COX-1) [47,48]. Apigenin, genistein, quercetin, catechin, and rhoifolin were capable of inhibiting TxB_2_ production in platelets in response to collagen but not arachidonic acid, which suggests that they do not directly inhibit COX-1 or thromboxane synthase (TS) but instead target upstream signalling molecules, which were not established yet [22]. Conversely, COX-1 has been suggested, via molecular docking experiments, to be an intracellular target for flavonoids such as those isolated by Wu et al. [36] as they are capable of forming hydrogen bonds with an arginine and/or a tyrosine residue in the gate of the active site and to sterically inhibit the conversion of AA to prostaglandin H_2_ (PGH_2_) by preventing its entry into the active site [28,36]. Furthermore, apigenin, genistein, quercetin, and catechin have been suggested to reversibly inhibit COX-1; however, the precise mechanism of action was not determined in this study [22]. In contrast to these results, Karlíčková et al. [49] used isolated ovine COX-1 to measure flavonoid-induced inhibition of prostaglandin H_2_ production and determined that 26 out of 28 flavonoids tested did not have a significant effect [49]. However, 100 μM genistein and 100 μM daidzein were significantly better than 100 μM acetylsalicylic acid (ASA) at inhibiting COX-1 activity in this assay, although in human PRP, they both were less potent and less efficacious than ASA. Furthermore, inhibition of human TS (required to convert PGH_2_ into TxA_2_) was achieved by 100 μM apigenin, 100 μM 7-hydroxyflavone, and 100 μM epicatechin; however, they were all significantly less potent than 1-benzylimidazole. All of the tested flavonoids in this study were able to significantly inhibit human platelet aggregation induced by U46619 [49]. Due to the supraphysiological concentrations required to achieve a significant inhibition, it is less likely that TS is a key mechanism for flavonoids-mediated platelet inhibition [49,50].

Moreover, nobiletin increased the levels of cyclic guanosine monophosphate (cGMP) from around 6 pM to 40 pM, which increases the activity of the inhibitory kinase, cGMP-dependent protein kinase (PKG). As expected, nobiletin increased the phosphorylation of vasodilator-stimulated phosphoprotein (VASP) at serine 239, which has been identified as the major site of PKG phosphorylation [19,51]. The pro-aggregatory ability of CRP-XL could be partially recovered by the inhibition of guanylyl cyclase or PKG (to approximately 60% of untreated). The cause of the increase in cGMP has been suggested to be due to the increased production of cGMP rather than decreased hydrolysis as tangeretin, a flavonoid with a similar structure, also increased the level of cGMP in the platelet cytosol; however, it was not due to the inhibition of cGMP phosphodiesterase as its activity was unaffected by the presence of tangeretin (Figure 3) [20].

Direct potentiation of adenylyl cyclase is another mechanism of action for flavonoid-mediated inhibition in platelets as cyclic adenosine monophosphate (cAMP) levels were observed to be increased in the presence of EGCG. Phosphorylation of VASP on a site specific for cAMP-dependent protein kinase (PKA) was also siginificantly increased, and the inhibitory effect was partially reversible by treatment with inhibitors of adenylyl cyclase. This was suggested to be due to the removal of PKA’s inhibitory phosphorylation of the inositol trisphosphate (IP_3_) receptor [23]. The specific location of flavonoid action is shown in Figure 3.

During intracellular signalling, several phosphorylation events occur. Interestingly, the phosphorylation of cytosolic proteins (Syk and Linker for Activation of T cells (LAT)) that are closely associated with GPVI-mediated signalling were unaffected by nobiletin or tangeretin treatment, which suggests that the ligation and activation of GPVI proceeds as normal into the LAT signalosome; however, signalling is then restricted to this area as phosphorylation of PLCγ2 and Akt (Protein Kinase B) was inhibited in a dose-dependent manner. Furthermore, whole cell tyrosine phosphorylation was largely unaffected by this flavonoid treatment. This strongly suggests that nobiletin may inhibits the function of phosphoinositide 3-kinase (PI3K) although the binding site and mechanism of action of this inhibition has yet to be fully elucidated [8,19,20]. Apigenin, genistein, and quercetin were shown to be inhibitors of multiple kinases involved in platelet activatory signalling pathways although the inhibition was not specific. These compounds inhibited Fyn, Lyn, Src, Syk, mitogen-activated protein kinase (MAPK)14, Akt1, Akt2, PI3Kβ, PI3Kγ, and PI3Kδ [21,38]. However, genistein had the weakest inhibitory effect of these three compounds on kinase activity. Quercetin also had inhibitory effects on different PKC isoforms (β1 and δ). Interestingly, rutin, a flavonoid that was not observed to affect platelet aggregation was able to mildly inhibit Src, Syk, PI3Kβ, PI3Kγ, and PI3Kδ. Furthermore, when whole cell tyrosine phosphorylation was examined, only apigenin and quercetin were able to significantly inhibit tyrosine phosphorylation [38]. Quercetin was found to inhibit the activity of Fyn, Syk, and PLCγ2 in the GPVI signalling complex although the target of action may be restricted to Fyn as this protein is found upstream from the other two in the signalling cascade [8,15,24]. Tamarixetin, a methoxylated metabolite of quercetin, inhibits tyrosine phosphorylation in platelets to a similar extent to quercetin (approximately 20% at 20 μM) [15]. However, tamarixetin is a more potent inhibitor of tyrosine phosphorylation on Syk. Quercetin-3′-sulphate (Q-3′-S) and quercetin-3-glucuronide (Q-3-G) do not significantly inhibit platelet tyrosine phosphorylation. Tamarixetin is as equally potent as quercetin in terms of PLCγ2 tyrosine phosphorylation but, unlike quercetin and Q-3′-S, cannot inhibit the kinase activity of Fyn [15]. Furthermore, these proteins seem like strong candidates for the site of flavonoid action as tyrosine phosphorylation is reduced in the platelets of volunteers who ingested Q-4-G with both Syk and PLCγ2 phosphorylation decreasing 120 min following ingestion [42]. Moreover, another flavonoid, chrysin, inhibits Syk, PLCγ2, and extracellular signal-regulated kinase (ERK)1/2 phosphorylation induced by collagen as well as inhibits PKC activity, a ubiquitous protein that is key for many intracellular signalling pathways [25,52].

### 2.3. Impact of Flavonoids on Granule Secretion in Platelets

P-Selectin exposure is a useful marker for platelet α-granule secretion [7], and adenosine triphosphate or serotonin release can signify dense granule secretion [10]. Together, these markers enable characterisation of the effects of flavonoids on platelet granule secretion. Nobiletin, tangeretin, and chrysin have been demonstrated to inhibit both α- and dense granule secretion [19,20,25]. Similarly, apigenin (100 μM; 65.4 ± 3.4% inhibition), genistein (100 μM; 57.5 ± 3.3% inhibition), luteolin (100 μM; 64.9 ± 6.4% inhibition), quercetin (200 μM; 56.7 ± 8.9% inhibition), catechin (500 μM; 61.7 ± 1.1% inhibition), rhoifolin (1 mM; 50 ± 2.2% inhibition), rutin (Figure 2C; 1 mM; 10.9 ± 2.8% inhibition), dimethylapigenin (Figure 2D; 25 μM; 29.6 ± 10.9% inhibition), and diosmetin (Figure 2E; 50 μM; 70.7 ± 9.5% inhibition) have been shown to inhibit dense granule secretion by a maximum of 65% (this level of inhibition by the most potent flavonoids was comparable to 1.1 mM ASA) [15,22]. The small degree of inhibition induced by catechin, rhoifolin, and rutin at such high concentrations implies that they may not be effective at inhibiting dense granule secretion under normal physiological conditions. Interestingly, quercetin has been demonstrated not to affect α-granule secretion induced by a selective PAR4 agonist in murine platelets following chronic treatment for one week even though dense granule secretion was affected (29.3 ± 12.5% release versus 55.0 ± 4.1% release in untreated samples) [39]. This is presumably due to differences in secretory mechanisms between α-granules and dense granules. Moreover, mouse platelets obtained from chronic quercetin-treated mice also exhibited a decreased dense granule secretion although α-granule secretion was unaffected [39].

### 2.4. Structural Considerations of Flavonoids

Due to the wide range of naturally occurring flavonoid structures, it is possible to investigate the effect of their functional groups on specific biological pathways. In general, the molecules that lacked efficacy possessed methoxy or large glycoside groups whereas the potent molecules possessed hydroxy groups that are capable of acting as hydrogen bond donors [22]. For example, apigenin, luteolin, rhoifolin, genistein, quercetin, and catechin were capable of inhibiting platelet aggregation induced by collagen, AA, or U46619 whereas rutin (possessing a disaccharide), dimethylapigenin (methylated at C6 and C8), and diosmetin (methoxylated at C4′) could not inhibit aggregation induced by AA or U46619 [22]. Despite their lower activites, the incorporation of methoxy groups may increase the ability of flavonoids to cross the cell membrane [15]. Moreover, the inhibitory potential of quercetin was strongly linked to its planar structure [15,24]. It should be noted that planar structures in flavonoids may suggest that they are packed together more efficiently, which decreases their solubility in water [53].

### 2.5. Effect of Flavonoids on Other Cells

Although most of the antiplatelet effects of flavonoids are due to direct effects on platelets, it has also been suggested that their effects can be due to paracrine effects on endothelial cells [37,54,55]. For example, pomegranate juice was seen to significantly increase the level of prostaglandin I_2_ (PGI_2_) (a potent platelet inhibitor [56]) in human plasma 2 h after consumption, but this effect was lost 6 h after ingestion [54].

Despite the promising potential of natural flavonoids, there are currently none routininely prescribed by practitioners in medical clinics for the treatment of thrombotic diseases. This is due to the issues described in the previous sections involving compound bioavailability, stability, and multiple cellular targets. In particular, the flavonoid concentrations achieved in the blood tend to be far less than their IC_50_ concentrations. 

## 3. Synthetic Flavonoids

Since natural flavonoids show promising but hampered potential for drug development, synthetic flavonoids have been developed by multiple laboratories to address the myriad of issues that are associated with natural flavonoids. The synthethic flavonoids have been developed primarily to overcome the issues raised in the previous sections regarding bioavailability (and interactions with plasma proteins) and stability. Many experiments have been conducted to determine the specific functional groups necessary for the beneficial antiplatelet effects that natural flavonoids exhibit.

### 3.1. Synthetic Flavonoids Affect Platelet Function

Chrysin is a naturally occurring flavonoid that is capable of inhibiting platelet activation in isolated platelets; however, its potency is significantly reduced when the experiment is repeated in PRP (in the presence of plasma proteins) [25,57]. In order to determine whether synthetic modifications to the structure of chrysin may alleviate the interference of plasma proteins, a library of compounds was synthesised through various modifications to the chemical structure of chrysin. For example, the conjugation of chrysin to ruthenium (Ru; Figure 2F) significantly increased the inhibitory effects of this molecule in PRP and whole blood [57]. Furthermore, a substitution of the carbonyl group with a thiocarbonyl group provides additional inhibitory effects and makes the compound more potent in the modulation of platelet function (e.g., 25 μM of chrysin induced approximately 25% inhibition in PRP compared to approximately 65% inhibition with 25 μM of Ru-thiochrysin) [57]. The efficacy of Ru-thiochrysin in isolated platelets is similar to chrysin, which suggests that the observed differences are due to the lack of binding to plasma proteins rather than to an increased binding capacity to or potency at effector molecules. The conjugation of flavonoids to metals such as ruthenium has been suggested to improve the molecule’s stability, membrane permeability, and enable more efficient binding to the target molecules [57,58]. It has also been suggested that conjugation to ruthenium would reduce side effects of compounds, and indeed, no toxic effects were seen in the mice treated with the Ru-conjugated compound [57].

Del Turco et al. [59] developed multiple synthetic 2,3-diphenyl-4*H*-pyrido(1,2-α)pyrimidin-4-one flavonoids and discovered that two of these compounds with multiple methoxy groups (Compound **6** (Figure 2G) and Compound **7** (Figure 2H)) were more potent than either quercetin or apigenin in response to the activation of PRP by collagen or U46619 [59].

The development of synthetic flavonoids enables modifications to the chemical structure in order to allow investigation of the effect of different functional groups on the antiplatelet ability of the compounds. F-1 (Figure 2I) is a synthetic flavonoid with a very basic flavonoid structure, which enables structure–activity relationships to be deduced [35]. Substitution of the carbonyl group with a thiocarbonyl group (TF-1, Figure 2I) broadened the inhibitory range of the compound to make it inhibitory at 25–100 μM in response to CRP-XL-induced isolated platelet aggregation, whereas substitution of the two hydroxyl groups with methoxy groups (CYC-1 (Figure 2I) and TCYC-1 (Figure 2I)) removed the inhibitory effects. These modifications were repeated with alternative B-ring structures such as replacing the phenyl B-ring with a thiofuran ring (F-2, TF-2, CYC-2, and TCYC-2 (Figure 2J)). Here, the responses were similar; however, the thiol version (TF-2) had reduced potency [35]. Substitution with a furan B-ring (F-3; Figure 2K) had little effect compared to the phenyl B-ring with inhibition remaining at approximately 50%; however, substitution of the B-ring with a pyridine B-ring (F-4; Figure 2L) removed all the anti-aggregatory effects induced by the synthetic flavonoids at the same concentrations [35]. The observed effects are likely due to alteration of the interaction of the synthetic flavonoids with target proteins as the hydroxyl groups act as hydrogen bond donors (with this interaction lost due to the substitution with a methoxy group), and the activity evoked by the B-ring was not affected by a change in the shape of the ring, but it was altered by a change in its chemistry. The loss of antiplatelet effects due to the addition of methoxy groups is concerning as extensive methoxylation has been suggested to protect compounds from metabolic changes that it would encounter if this compound was provided as a prophylactic treatment [60]. Other studies on the effects of functional groups have, however, suggested that the presence of hydroxyl groups at C3 and C6 increases the potency and efficacy [61]. Interestingly, these two studies found that the presence of a carbon–carbon double bond between C2 and C3 leads to decreased inhibition when methoxy groups are substituted onto the B-ring although the inverse is true when there is no double bond at this location [35,61]. The effect of methoxylation appears to be complicated by the multiple mechanisms of action presented by flavonoids, which hampers the construction of a comprehensive set of rules, and therefore, further research on the impact of methoxylation on flavonoids should be performed.

Quercetin’s inhibitory potential can be increased via the addition of an acyl chain between 3^rd^ and 4^th^ carbon atoms (Figure 2M), which helps disrupt molecular packing [53]. Acylation of quercetin decreased ADP-, AA-, or platelet activating factor (PAF)-induced platelet aggregation more than the natural unacylated form (67.8% (quercetin-3-*O*-propionate) and 52.7% (quercetin-3-*O*-butyrate) versus 43.5% inhibition for quercetin), potentially due to the change in molecular packing [53]. PMQ inhibited U46619- and ADP-induced platelet aggregation [40].

3′,4′-Dihydroxyflavonol (DiOHF; a modified form of quercetin; Figure 2N) decreases murine PRP aggregation in response to a PAR4 agonist whether administered in a single IV bolus (47.0 ± 4.0% versus 57.6 ± 6.1%) or IP over the period of a week (50.4 ± 6.6% versus 73.4 ± 4.6%) [39,62]. Synthetic flavonoids that have been proposed to act as TP receptor antagonists are ineffective at inhibiting thrombin-induced platelet aggregation [59]. Despite Ru-thiochrysin imparting a greater effect on plasma proteins *ex vivo*, no significant difference between the tail bleeding times of mice treated with chrysin or Ru-thiochrysin was seen at the same concentration, which also emphasised this as a safer compound for therapeutic applications [57]. Additionally, DiOHF was effective at maintaining blood flow in a ferric chloride-induced arterial injury model following acute administration; however, the significant impact was lost when the compound was supplied IP for a week, even though quercetin was still effective [39]. More studies are required in order to underpin the actions of synthetic flavonoids in the modulation of platelet function, thrombosis, and haemostasis under *in vivo* settings.

### 3.2. Impact of Synthetic Flavonoids on Granule Secretion

Both chrysin and Ru-thiochrysin inhibit dense- and α-granule secretion; however, Ru-thiochrysin inhibits P-selectin exposure more than chrysin, yet there were no significant differences between their effects on dense granule secretion [57]. Other synthetic flavonoids such as 2,3-diphenyl-4*H*-pyrido(1,2-α)pyrimidin-4-one flavonoids decrease α-granule secretion induced by U46619 [59]. Conversely, PAR4-mediated dense granule secretion was inhibited by DiOHF but with the same efficacy as quercetin; meanwhile, α-granule secretion was unaffected by either compound [39].

### 3.3. Synthetic Flavonoids Exert Selective Effects

Conjugation of ruthenium to chrysin (Ru-thiochrysin) was also significantly more effective at inhibiting calcium mobilisation, fibrinogen binding, and clot retraction in PRP whereas there was no difference between the two compounds in isolated platelets [57].

Limited studies have investigated the ligation of synthetic flavonoids to specific molecular targets on the surface of platelets. Naturally occurring flavonoids have been suggested to have extracellular targets and 2,3-diphenyl-4H-pyrido(1,2-α)pyrimidin-4-one flavonoids, like apigenin, have been shown to antagonise TP receptor [22,59].

An immunoblotting analysis of Ru-thiochrysin and chrysin on the phosphorylation of focal adhesion kinase (FAK), Akt, and Src showed a decrease in the phosphorylation; however, the target protein(s) of chrysin and its synthetic derivatives were not fully elucidated [57].

### 3.4. Synthetic Flavonoids Display Improved Bioavailability Compared to Their Natural Counterparts

Synthetic modifications of flavonoids are required to increase the therapeutic potential of flavonoids as natural flavonoids have non-ideal bioavailabilities. For example, it was calculated in rodents that to achieve an effective plasma concentration (of approximately 50 µM), 100 mg/kg of tangeretin or nobiletin would be necessary, which would equate to >7 g/day in human adults [63]. When the flavone contents of kumquats (21.87 mg/100 g edible portion), lemons (1.90 mg/100 g edible portion), and oranges (0.19 mg/100 g edible portion) are taken into account, the unfeasible nature of this becomes clear [27]. Furthermore, in humans, quercetin levels were shown to peak at 30 min after ingestion, to then decline to approximately 20% of the peak after two hours, and then to plateau for the following 32 h. However, quercetin was still able to inhibit platelet activation in response to low levels of collagen at least up to two hours after ingestion [42]. Naturally occurring flavonoids have poor bioavailability due to diminished absorption in the gastrointestinal tract [64]. Flavonoid insolubility is partially related to its supramolecular structure, and their shape facilitates tight packing, which makes it difficult for individual molecules to dissociate [53]. Acylation of the functional groups of flavonoids was proposed to increase the bioavailability of quercetin, and it was found that acylation could increase water solubility and lipophilicity but that the magnitude of the effect varied based on the length of the acyl chain (potentially due to changes in the packing of quercetin) [53]. Quercetin-3-*O*-propionate (16.27 μg/mL) was the most soluble in water when compared to quercetin-3-*O*-butyrate (9.36 μg/mL), quercetin (1.98 μg/mL), and quercetin-3-*O*-valerate (1.07 μg/mL) [53]. A 3 or 4 carbon acyl chain was found to be the most effective at inhibiting platelet aggregation compared to quercetin for a range of platelet agonists both *in vitro* and *in vivo*. Longer acyl chains had a negative impact and reduced hydrosolubility and potency [53]. A balance must be struck, however, between hydrophilicity and lipophilicity as increased water solubility may lead to a decrease in the passive absorption of the flavonoid in the intestine [27]. Mild hydrophilic qualities might help prevent aggregate formation and, therefore, aid in its absorption across the intestinal endothelium.

Synthetic flavonoids could also be produced to resist metabolism *in vivo*. This is a key consideration as it has been shown that platelets can metabolise flavonoids on their own [16]. For example, the naturally occurring flavonoid quercetin was discovered to be a platelet inhibitor; however, its metabolites, tamarixetin, Q-3′-S, and Q-3-G were found to be less potent [15]. A synthetic flavonoid that is resistant to this metabolism may provide a longer-acting and more potent drug. Conversely, metabolism of quercetin in the human body increases its ability to cross plasma membranes and enter cells [15]. Equol is another flavonoid metabolite (from daidzein) and, as mentioned previously, is capable of inhibiting platelet function [32]. Interestingly, the only difference between daidzein and equol is the lack of a carbonyl group at carbon-4, which suggests that the oxygen atom promotes an unfavourable interaction with the TP receptor.

Additionally, most studies examining natural flavonoids utilise isolated platelet preparations to mitigate the influence of plasma proteins [20,57]. However, conjugation of flavonoids with metals has been proposed to decrease interactions between the flavonoid molecule and proteins present in the blood plasma and, therefore, to significantly increase the efficacy of the flavonoid in more physiologically relevant conditions [57]. It has been demonstrated by work conducted in our laboratory that these flavonoid-metal conjugates can significantly increase the inhibitory potential of flavonoids [57].

Thus far, characterisation of flavonoid efficacy has mainly been limited to adjusting the functional groups on a flavonoid backbone and testing a wide range of compounds on platelet aggregation without examining the molecular targets involved or determining why the specific functional groups are effective. However, they still provide a valuable insight into potential modifications that could be used in future drug design [35,61]. This information could then be used in future studies for *in silico* molecular docking analysis coupled with functional screening of predicted compounds.

## 4. Conclusions

Platelet activation can be inhibited by flavonoids via multiple mechanisms; however, naturally occurring flavonoids raise challenges with reduced bioavailability and metabolism to form less efficacious metabolites. Synthetic flavonoids enable the tailoring of flavonoid structures into better therapeutic agents. However, the benefits observed for currently used synthetic flavonoids *ex vivo* have yet to be replicated *in vivo*. Indeed, flavonoids provide a good template for future drug design, but further experiments, particularly investigating the structure–activity relationships of functional groups in more detail and the specific molecular targets of flavonoids, must be conducted for the potential of flavonoids as therapeutic agents to be realised. Although studies on synthetic flavonoids are limited, they provide a robust starting point to take this research further in order to develop better antithrombotic agents using flavonoids as a basis.

Further development of synthetic flavonoids that are more specific for effector proteins would provide a greater opportunity for achieving therapeutic agents that are as effective as current treatments but without the unwanted and dangerous side effects currently encountered. If flavonoid molecules could be biased towards the inhibition of specific sectors of platelet activity, then this could improve the tolerance of drugs. These novel compounds could be designed to only mildly inhibit collagen-induced platelet activation but to strongly inhibit platelet activation induced by secondary activators such as ADP and TxA_2_ to mitigate the size of the thrombus whilst still enabling platelets to respond to damage at the sites of injury. Flavonoids also possess the ability to influence the activity of other cell types such as endothelial cells and, therefore, may provide additional inhibitory effects not limited to direct actions on platelets.
